# 3-(2*H*-Tetra­zol-5-yl)pyridinium trifluoro­acetate

**DOI:** 10.1107/S1600536809033054

**Published:** 2009-09-09

**Authors:** Li Zhang

**Affiliations:** a Ordered Matter Science Research Center, College of Chemistry and Chemical Engineering, Southeast University, Nanjing 210096, People’s Republic of China

## Abstract

In the cation of the title compound, C_6_H_6_N_5_^+^·CF_3_COO^−^, the pyridine and tetra­zole rings are nearly coplanar, making a dihedral angle of 2.49 (19)°. In the crystal, the cations and anions are connected by inter­molecular N—H⋯O and N—H⋯(F,O) hydrogen bonds, forming centrosymmetric [2 + 2] aggregates, which stack along the *a* axis.

## Related literature

For the applications of metal-organic coordination compounds, see: Fu *et al.* (2007 ▶); Huang *et al.* (1999 ▶); Fu & Xiong (2008 ▶); Liu *et al.* (1999 ▶); Xie *et al.* (2003 ▶); Zhang *et al.* (2000 ▶, 2001 ▶). For tetra­zole derivatives, see: Fu *et al.* (2008 ▶); Wang, *et al.* (2005 ▶).
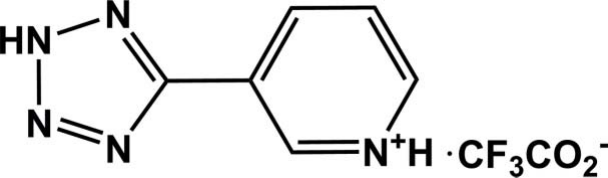


## Experimental

### 

#### Crystal data

C_6_H_6_N_5_^+^·C_2_F_3_O_2_^−^
*M* *_r_* = 261.18
Triclinic, 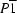

*a* = 4.8564 (10) Å
*b* = 9.5989 (19) Å
*c* = 11.917 (2) Å
α = 90.02 (3)°
β = 101.34 (3)°
γ = 98.55 (3)°
*V* = 538.35 (19) Å^3^
*Z* = 2
Mo *K*α radiation
μ = 0.15 mm^−1^
*T* = 298 K
0.30 × 0.25 × 0.20 mm

#### Data collection

Rigaku Mercury2 diffractometer
Absorption correction: multi-scan (*CrystalClear*; Rigaku, 2005 ▶) *T*_min_ = 0.96, *T*_max_ = 1.00 (expected range = 0.931–0.970)
5516 measured reflections
2434 independent reflections
1074 reflections with *I* > 2σ(*I*)
*R*_int_ = 0.061

#### Refinement

*R*[*F* ^2^ > 2σ(*F* ^2^)] = 0.075
*wR*(*F* ^2^) = 0.241
*S* = 0.93
2434 reflections
163 parameters
H-atom parameters constrained
Δρ_max_ = 0.27 e Å^−3^
Δρ_min_ = −0.26 e Å^−3^

### 

Data collection: *CrystalClear* (Rigaku, 2005 ▶); cell refinement: *CrystalClear*; data reduction: *CrystalClear*; program(s) used to solve structure: *SHELXS97* (Sheldrick, 2008 ▶); program(s) used to refine structure: *SHELXL97* (Sheldrick, 2008 ▶); molecular graphics: *SHELXTL* (Sheldrick, 2008 ▶); software used to prepare material for publication: *SHELXTL*.

## Supplementary Material

Crystal structure: contains datablocks I, global. DOI: 10.1107/S1600536809033054/su2125sup1.cif

Structure factors: contains datablocks I. DOI: 10.1107/S1600536809033054/su2125Isup2.hkl

Additional supplementary materials:  crystallographic information; 3D view; checkCIF report

## Figures and Tables

**Table 1:** Hydrogen-bond geometry (Å, °)

*D*—H⋯*A*	*D*—H	H⋯*A*	*D*⋯*A*	*D*—H⋯*A*
N1—H1*A*⋯O2^i^	0.86	1.79	2.651 (4)	176
N4—H4*A*⋯O1^ii^	0.86	1.87	2.716 (4)	167
N4—H4*A*⋯F3^ii^	0.86	2.53	3.053 (4)	120

Symmetry codes: (i) 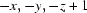; (ii) 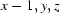.

**Fig. 1.**
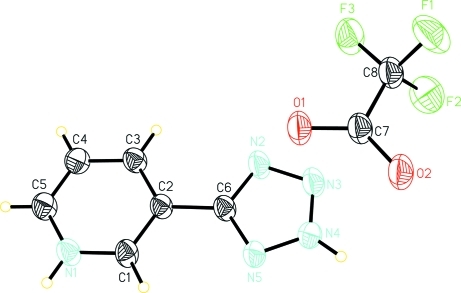
A view of themolecular structure of the title compound, showing the displacement ellipsoids drawn at the 30% probability level.

**Fig. 2.**
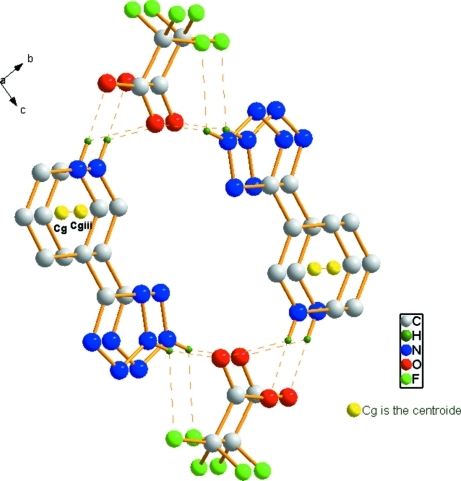
The crystal packing of the title compound, viewed approximately along the a axis, showing the π···π, N—H···O and N—H···F interactions as dotted lines [see Table 1 for details; H atoms not involved in hydrogen bonding have been omitted for clarity].

## supplementary crystallographic information

### Comment

The construction of metal-organic coordination compounds has attracted much attention owing to their potential functions, such as permittivity, fluorescence, magnetism and optical properties. (Fu *et al.*, 2007; Huang *et al.*, 1999; Fu & Xiong, 2008; Liu *et al.*, 1999; Xie *et al.*, 2003; Zhang *et al.*, 2001; Zhang *et al.*, 2000). Tetrazole derivatives are a class of excellent ligands because of their multiple coordination modes and for the construction of novel metal-organic frameworks. (Wang, *et al.* 2005; Fu *et al.*, 2008). We report herein on the crystal structure of the title compound, 3-(2*H*-tetrazol-5-yl)pyridinium Trifluoroacetate.

In the title compound (Fig. 1), the pyridine N atoms are protonated. The pyridine and tetrazole rings are nearly coplanar and twisted with respect to one another by only 2.49 (19) °. The geometric parameters of the tetrazole rings are comparable to those in related molecules (Wang, *et al.*, 2005; Fu *et al.*, 2008).

The crystal packing is stabilized by intermolecular N—H···O and N—H···F hydrogen bonds forming centrosymmetric [2 + 2] aggregates, which stack along the *a* axis (Fig. 2 and Table 1). The pyridine rings [*Cg*···*Cg^i^*] of neighbouring cation systems are separated by 4.856 (2) Å [*Cg* is the centroid of the pyridine rings, symmetry code (i) = *x* + 1, *y*, *z*].

### Experimental

Isonicotinonitrile (30 mmol), NaN_3_ (45 mmol), NH_4_Cl (33 mmol) and DMF (50 ml) were added in a flask under a nitrogen atmosphere and the mixture stirred at 383 K for 20 h. The resulting solution was then poured into ice-water (100 ml), and a white solid was obtained after adding HCl (6 *M*) and adjusting the pH = 6. The precipitate was filtered off and washed with distilled water. Colourless block-shaped crystals, suitable for X-ray analysis, were obtained from the crude product by slow evaporation of an ethanol/CF_3_COOH [10:1 *v*/*v*] solution.

### Refinement

The H-atoms were included in calculated positions and treated as riding atoms: N–H = 0.86 Å, C–H = 0.93 Å, with *U*_iso_(H) = 1.2U_eq_(parent N- or C-atom).

### Crystal data

**Table:**

C_6_H_6_N_5_^+^·C_2_F_3_O_2_^−^	*Z* = 2
*M**_r_* = 261.18	*F*(000) = 264
Triclinic, *P*1	*D*_x_ = 1.611 Mg m^−^^3^
Hall symbol: -P 1	Mo *K*α radiation, λ = 0.71073 Å
*a* = 4.8564 (10) Å	Cell parameters from 1074 reflections
*b* = 9.5989 (19) Å	θ = 3.5–27.5°
*c* = 11.917 (2) Å	µ = 0.15 mm^−^^1^
α = 90.02 (3)°	*T* = 298 K
β = 101.34 (3)°	Block, colorless
γ = 98.55 (3)°	0.30 × 0.25 × 0.20 mm
*V* = 538.35 (19) Å^3^	

### Data collection

**Table:**

Rigaku Mercury2 diffractometer	2434 independent reflections
Radiation source: fine-focus sealed tube	1074 reflections with *I* > 2σ(*I*)
graphite	*R*_int_ = 0.061
Detector resolution: 13.6612 pixels mm^-1^	θ_max_ = 27.5°, θ_min_ = 3.5°
CCD profile fitting scans	*h* = −6→6
Absorption correction: multi-scan (*CrystalClear*; Rigaku, 2005)	*k* = −12→12
*T*_min_ = 0.96, *T*_max_ = 1.00	*l* = −15→15
5516 measured reflections	

### Refinement

**Table:**

Refinement on *F*^2^	Primary atom site location: structure-invariant direct methods
Least-squares matrix: full	Secondary atom site location: difference Fourier map
*R*[*F*^2^ > 2σ(*F*^2^)] = 0.075	Hydrogen site location: inferred from neighbouring sites
*wR*(*F*^2^) = 0.241	H-atom parameters constrained
*S* = 0.93	*w* = 1/[σ^2^(*F*_o_^2^) + (0.1213*P*)^2^] where *P* = (*F*_o_^2^ + 2*F*_c_^2^)/3
2434 reflections	(Δ/σ)_max_ < 0.001
163 parameters	Δρ_max_ = 0.27 e Å^−^^3^
0 restraints	Δρ_min_ = −0.26 e Å^−^^3^

### Special details

**Table:**

Geometry. All e.s.d.'s (except the e.s.d. in the dihedral angle between two l.s. planes) are estimated using the full covariance matrix. The cell e.s.d.'s are taken into account individually in the estimation of e.s.d.'s in distances, angles and torsion angles; correlations between e.s.d.'s in cell parameters are only used when they are defined by crystal symmetry. An approximate (isotropic) treatment of cell e.s.d.'s is used for estimating e.s.d.'s involving l.s. planes.
Refinement. Refinement of *F*^2^ against ALL reflections. The weighted *R*-factor *wR* and goodness of fit *S* are based on *F*^2^, conventional *R*-factors *R* are based on *F*, with *F* set to zero for negative *F*^2^. The threshold expression of *F*^2^ > σ(*F*^2^) is used only for calculating *R*-factors(gt) *etc*. and is not relevant to the choice of reflections for refinement. *R*-factors based on *F*^2^ are statistically about twice as large as those based on *F*, and *R*- factors based on ALL data will be even larger.

### Fractional atomic coordinates and isotropic or equivalent isotropic displacement parameters (Å^2^)

**Table:**

	*x*	*y*	*z*	*U*_iso_*/*U*_eq_	
C1	0.1751 (8)	0.1275 (4)	0.6775 (3)	0.0573 (10)	
H1	0.0072	0.0667	0.6502	0.069*	
C2	0.2548 (7)	0.2409 (3)	0.6141 (3)	0.0513 (9)	
C3	0.5092 (7)	0.3283 (4)	0.6578 (3)	0.0584 (9)	
H3	0.5695	0.4055	0.6169	0.070*	
C4	0.6729 (8)	0.3005 (4)	0.7619 (3)	0.0643 (10)	
H4	0.8421	0.3593	0.7912	0.077*	
C5	0.5847 (8)	0.1867 (4)	0.8213 (3)	0.0652 (11)	
H5	0.6946	0.1666	0.8908	0.078*	
C6	0.0725 (7)	0.2678 (4)	0.5049 (3)	0.0532 (9)	
N1	0.3391 (6)	0.1041 (3)	0.7789 (2)	0.0632 (9)	
H1A	0.2837	0.0335	0.8177	0.076*	
N2	0.1380 (7)	0.3773 (3)	0.4385 (3)	0.0715 (10)	
N3	−0.0790 (7)	0.3696 (4)	0.3500 (3)	0.0745 (10)	
N4	−0.2582 (6)	0.2577 (3)	0.3670 (3)	0.0663 (9)	
H4A	−0.4163	0.2309	0.3202	0.080*	
N5	−0.1752 (6)	0.1894 (3)	0.4621 (2)	0.0628 (9)	
O1	0.2404 (5)	0.1352 (3)	0.2369 (2)	0.0703 (8)	
F1	0.2471 (6)	0.2764 (3)	−0.0109 (2)	0.1132 (11)	
F2	−0.0045 (6)	0.3887 (3)	0.0715 (2)	0.1058 (10)	
F3	0.4227 (5)	0.3786 (3)	0.1514 (2)	0.1008 (10)	
C7	0.0877 (8)	0.1692 (4)	0.1485 (3)	0.0590 (10)	
C8	0.1930 (8)	0.3036 (4)	0.0912 (3)	0.0673 (11)	
O2	−0.1502 (6)	0.1066 (3)	0.0989 (2)	0.0862 (10)	

### Atomic displacement parameters (Å^2^)

**Table:**

	*U*^11^	*U*^22^	*U*^33^	*U*^12^	*U*^13^	*U*^23^
C1	0.070 (2)	0.055 (2)	0.0397 (19)	0.0043 (18)	−0.0015 (17)	−0.0042 (16)
C2	0.059 (2)	0.052 (2)	0.0383 (18)	0.0025 (17)	0.0044 (15)	−0.0029 (14)
C3	0.068 (2)	0.058 (2)	0.045 (2)	0.0004 (18)	0.0074 (17)	−0.0002 (15)
C4	0.066 (2)	0.070 (3)	0.048 (2)	−0.0051 (19)	0.0014 (17)	−0.0073 (18)
C5	0.072 (2)	0.071 (3)	0.045 (2)	0.001 (2)	0.0020 (18)	−0.0044 (18)
C6	0.065 (2)	0.053 (2)	0.0375 (18)	0.0037 (17)	0.0048 (16)	−0.0015 (15)
N1	0.077 (2)	0.0609 (19)	0.0447 (18)	−0.0009 (16)	0.0026 (15)	0.0043 (13)
N2	0.080 (2)	0.075 (2)	0.0441 (18)	−0.0107 (17)	−0.0072 (16)	0.0092 (15)
N3	0.083 (2)	0.082 (2)	0.0460 (19)	−0.0026 (19)	−0.0043 (16)	0.0089 (15)
N4	0.0649 (19)	0.077 (2)	0.0476 (19)	−0.0049 (17)	0.0008 (15)	−0.0014 (15)
N5	0.0648 (19)	0.072 (2)	0.0421 (17)	−0.0028 (16)	−0.0021 (14)	0.0045 (14)
O1	0.0763 (17)	0.0729 (18)	0.0502 (15)	0.0032 (13)	−0.0092 (13)	0.0106 (12)
F1	0.149 (3)	0.127 (2)	0.0600 (17)	−0.014 (2)	0.0378 (17)	−0.0015 (14)
F2	0.118 (2)	0.0849 (18)	0.114 (2)	0.0232 (16)	0.0168 (17)	0.0347 (15)
F3	0.109 (2)	0.0907 (18)	0.0755 (17)	−0.0302 (15)	−0.0140 (15)	0.0169 (13)
C7	0.069 (2)	0.057 (2)	0.044 (2)	0.0018 (18)	−0.0005 (17)	0.0020 (16)
C8	0.075 (3)	0.069 (3)	0.047 (2)	−0.005 (2)	−0.0023 (19)	0.0037 (18)
O2	0.0905 (19)	0.084 (2)	0.0585 (17)	−0.0209 (16)	−0.0200 (14)	0.0166 (14)

### Geometric parameters (Å, °)

**Table:**

C1—N1	1.349 (4)	C6—N2	1.354 (4)
C1—C2	1.376 (5)	N1—H1A	0.8600
C1—H1	0.9300	N2—N3	1.329 (4)
C2—C3	1.393 (4)	N3—N4	1.321 (4)
C2—C6	1.470 (4)	N4—N5	1.333 (4)
C3—C4	1.384 (5)	N4—H4A	0.8600
C3—H3	0.9300	O1—C7	1.237 (4)
C4—C5	1.363 (5)	F1—C8	1.328 (4)
C4—H4	0.9300	F2—C8	1.336 (5)
C5—N1	1.337 (4)	F3—C8	1.313 (4)
C5—H5	0.9300	C7—O2	1.253 (4)
C6—N5	1.327 (4)	C7—C8	1.528 (5)
			
N1—C1—C2	120.4 (3)	C5—N1—C1	122.3 (3)
N1—C1—H1	119.8	C5—N1—H1A	118.8
C2—C1—H1	119.8	C1—N1—H1A	118.8
C1—C2—C3	117.8 (3)	N3—N2—C6	105.9 (3)
C1—C2—C6	120.2 (3)	N4—N3—N2	105.4 (3)
C3—C2—C6	122.0 (3)	N3—N4—N5	114.9 (3)
C4—C3—C2	120.3 (3)	N3—N4—H4A	122.5
C4—C3—H3	119.9	N5—N4—H4A	122.5
C2—C3—H3	119.9	C6—N5—N4	100.9 (3)
C5—C4—C3	119.7 (3)	O1—C7—O2	127.7 (3)
C5—C4—H4	120.2	O1—C7—C8	117.9 (3)
C3—C4—H4	120.2	O2—C7—C8	114.3 (3)
N1—C5—C4	119.6 (3)	F3—C8—F1	107.4 (3)
N1—C5—H5	120.2	F3—C8—F2	106.4 (3)
C4—C5—H5	120.2	F1—C8—F2	105.3 (3)
N5—C6—N2	113.0 (3)	F3—C8—C7	114.1 (3)
N5—C6—C2	123.8 (3)	F1—C8—C7	111.9 (3)
N2—C6—C2	123.2 (3)	F2—C8—C7	111.3 (3)
			
N1—C1—C2—C3	0.7 (5)	C2—C6—N2—N3	−178.0 (3)
N1—C1—C2—C6	−178.7 (3)	C6—N2—N3—N4	−0.5 (4)
C1—C2—C3—C4	−0.3 (5)	N2—N3—N4—N5	0.2 (5)
C6—C2—C3—C4	179.0 (3)	N2—C6—N5—N4	−0.6 (4)
C2—C3—C4—C5	0.4 (6)	C2—C6—N5—N4	178.1 (3)
C3—C4—C5—N1	−0.9 (6)	N3—N4—N5—C6	0.2 (4)
C1—C2—C6—N5	1.4 (5)	O1—C7—C8—F3	−8.1 (5)
C3—C2—C6—N5	−178.0 (3)	O2—C7—C8—F3	171.3 (4)
C1—C2—C6—N2	179.9 (3)	O1—C7—C8—F1	114.1 (4)
C3—C2—C6—N2	0.6 (6)	O2—C7—C8—F1	−66.5 (5)
C4—C5—N1—C1	1.2 (6)	O1—C7—C8—F2	−128.5 (4)
C2—C1—N1—C5	−1.1 (5)	O2—C7—C8—F2	50.9 (5)
N5—C6—N2—N3	0.7 (4)		

### Hydrogen-bond geometry (Å, °)

**Table:**

*D*—H···*A*	*D*—H	H···*A*	*D*···*A*	*D*—H···*A*
N1—H1A···O2^i^	0.86	1.79	2.651 (4)	176
N4—H4A···O1^ii^	0.86	1.87	2.716 (4)	167
N4—H4A···F3^ii^	0.86	2.53	3.053 (4)	120

Symmetry codes: (i) −*x*, −*y*, −*z*+1; (ii) *x*−1, *y*, *z*.
